# Metabolomic profiling of follicular fluid reveals unique pathways in endometriosis and infertility etiologies: a pilot study

**DOI:** 10.7717/peerj.20786

**Published:** 2026-02-10

**Authors:** Ping Yu, Dandan Chen, Deshen Han, Xin Jin, Yuhong Li, Fu Wei, Yun Zhang

**Affiliations:** 1 Center of Reproductive Medicine, Women’s Hospital of Jiangnan University, Wuxi Maternity and Child Health Care Hospital, Wuxi, China; 2 Wuxi School of Medicine, Jiangnan University, Wuxi, China; 3 Center of Reproductive Medicine, Guigang City People’s Hospital, Guigang, China

**Keywords:** Infertility etiology, Follicular fluid metabolomics, *In vitro* fertilization (IVF), Follicular fluid follicular development rate (FDR), Biomarkers

## Abstract

**Purpose:**

This study investigates metabolic profiles in follicular fluid of patients with endometriosis (EM), polycystic ovary syndrome (PCOS), tubal blockage (TB), and unexplained infertility (UEI), assessing their associations with follicular development and *in vitro* fertilization (IVF) outcomes. It aims to identify metabolic alterations and potential biomarkers for EM diagnosis and personalized reproductive strategies.

**Methods:**

Follicular fluid samples were collected from 12 infertility patients (3 EM, 3 PCOS, 3 TB, and 3 UEI) undergoing IVF. Metabolomic profiling was performed using liquid chromatography-mass spectrometry (LC-MS), followed by pathway enrichment analysis to identify key metabolic pathways. Statistical analyses were conducted to compare metabolic profiles across groups, assess correlations with follicular development rate (FDR), and evaluate potential biomarkers for EM diagnosis.

**Results:**

EM patients showed significant metabolic changes, including reduced steroid biosynthesis and elevated thiamine metabolism metabolites, linked to lower FDR. Oxidative stress markers (3-chloro-L-tyrosine, 8-oxoerythraline) were elevated and negatively correlated with FDR. A predictive model identified D-mannosamine, D-galacturonic acid, and 3-chloro-L-tyrosine as potential EM biomarkers with high diagnostic accuracy.

**Conclusion:**

This study reveals distinct metabolic disruptions in the follicular fluid of EM patients, particularly in steroid biosynthesis and thiamine metabolism pathways, which are linked to impaired follicular development. The identification of specific metabolites as potential biomarkers for EM provides a foundation for developing diagnostic approaches that minimize the need for additional invasive procedures and support personalized assisted reproductive technology (ART) strategies. This pilot study requires further validation to confirm these findings and translate them into clinical practice.

## Introduction

Infertility affects 10% to 15% of couples of reproductive age worldwide, with its prevalence increasing due to lifestyle and environmental factors, causing significant psychological and economic burdens (Fu et al., 2023). Assisted reproductive technology (ART), particularly *in vitro* fertilization (IVF), offers an effective treatment option for infertile couples (Boedt et al., 2021; Roomaney et al., 2024). During IVF, gonadotropins stimulate oocyte maturation within the ovarian follicles, where follicular fluid provides a critical microenvironment for oocyte growth and development. Follicular fluid, rich in metabolites, hormones, and growth factors, holds the key to understanding and enhancing IVF outcomes (Luti et al., 2020).

Follicular fluid metabolites, which reflect the biochemical environment essential for oocyte development, have been closely associated with IVF outcomes (Ban et al., 2021; Revelli et al., 2009). For instance, elevated levels of pyruvate and lactate are linked to both improved oocyte quality and higher fertilization rates (Xiong et al., 2024). Amino acids such as glutamine and alanine support redox balance and oocyte maturation while promoting early embryo development (Chen et al., 2018; Seli et al., 2014; Zhao et al., 2023). Conversely, increased oxidative stress markers like malondialdehyde (MDA) are associated with diminished oocyte quality, lower implantation success, and even reduced clinical pregnancy rates (Chen, Yang & Zhang, 2023). Additionally, imbalances in specific metabolites, such as elevated homocysteine levels, have been linked to an increased risk of miscarriage (Cavallé-Busquets et al., 2020). However, similar to the views of researchers such as Timothy Bracewell-Milnes (Bracewell-Milnes et al., 2017), this study believes that systemic metabolic changes driven by different infertility etiologies likely shape the composition of follicular fluid, warranting further exploration.

Female infertility is influenced by conditions such as ovarian endometriosis, polycystic ovary syndrome (PCOS), and tubal obstruction, which disrupt systemic metabolic processes and alter the metabolic composition of follicular fluid, as evidenced by emerging studies (Castiglione Morelli et al., 2019; Pandey, 2024; Shi et al., 2024; Zhang et al., 2025). These alterations have been linked to IVF outcomes, though findings are sometimes inconsistent. For instance, ovarian endometriosis has been associated with increased oxidative stress and abnormal follicular fluid levels of glutathione and lipid peroxides, indicating disruptions in energy metabolism and redox balance (Lu et al., 2023). Similarly, PCOS, characterized by hyperandrogenism and insulin resistance, shows dysregulated glucose and lipid metabolism, with altered levels of free fatty acids and amino acids in follicular fluid that affect oocyte quality (Dai et al., 2024). Tubal blockage (TB) elevates inflammation-related metabolites, including cytokines and reactive oxygen species, modifying the follicular environment (Volodarsky-Perel, Buckett & Tulandi, 2019). However, most studies have focused on comparing these conditions with control groups, leaving direct comparisons between different infertility etiologies largely unexplored. Moreover, little attention has been paid to how these conditions affect follicular development, a critical determinant of oocyte maturation and IVF success, underscoring the need for further research. Building on the understanding of systemic metabolic disruptions caused by conditions such as endometriosis (EM), PCOS, and TB, this study utilizes metabolomics to investigate how these alterations manifest in follicular fluid and their effects on follicle development and IVF success. By characterizing these metabolic profiles, we seek to deepen the understanding of these infertility-related conditions, identify potential metabolic biomarkers, and provide a foundation for personalized ART strategies to enhance IVF outcomes.

## Methods

### Study design

This study included female infertility patients undergoing *in vitro* fertilization and embryo transfer (IVF-ET) at Wuxi Maternity and Child Health Hospital, comprising three patients with endometriosis (EM), three with polycystic ovary syndrome (PCOS), three with tubal obstruction (TB), and three with unexplained infertility (UEI). EM patients were diagnosed by laparoscopy, with clinical features like pelvic pain and infertility, excluding severe comorbidities (*e.g.*, diabetes, cardiovascular disease). PCOS patients met Rotterdam criteria (at least two of: oligo/anovulation, hyperandrogenism, polycystic ovaries on ultrasound), with EM ruled out *via* history and imaging and severe metabolic disorders (*e.g.*, body mass index (BMI) > 35 kg/m2) excluded. TB was confirmed by hysterosalpingography (HSG) or laparoscopy as bilateral tubal blockage, excluding hydrosalpinx (*via* ultrasound/HSG), with EM and reproductive anomalies ruled out by laparoscopy or imaging. UEI patients had no identifiable cause after comprehensive evaluation (semen analysis, ovulation monitoring, HSG, hormone levels), with EM, PCOS, and tubal pathology excluded by history, imaging, or laparoscopy.

Before the initiation of treatment, the research team provided all participants with detailed information regarding the study’s purpose, procedures, potential risks, and benefits. After ensuring that the patients fully understood the study, each participant voluntarily signed an informed consent form. Additionally, this study was reviewed and approved by the Ethics Committee of Wuxi Maternity and Child Health Hospital (Approval No.: 2023-01-1031-42).

### Sample collection

Follicular fluid samples were collected from all participants undergoing controlled ovarian hyperstimulation (COH) as part of their *in vitro* fertilization (IVF) treatment at Wuxi Maternity and Child Health Hospital. COH was conducted using a gonadotropin-releasing hormone (GnRH) antagonist protocol. Stimulation began on day 2 or 3 of the menstrual cycle with recombinant follicle-stimulating hormone (rFSH) at an initial dose of 150–300 IU/day, adjusted according to patient age, body mass index (BMI), antral follicle count (AFC), and anti-Mļlerian hormone (AMH) levels. Transvaginal ultrasound monitoring and serum estradiol (E2) assessments were performed every 2–3 days starting on day 5 of stimulation to evaluate follicular growth and adjust rFSH dosage if needed. Once the dominant follicle reached a diameter of 14 mm, (flexible stimulation protocol) or from 6 th day of hormonal stimulation (fixed protocol) a GnRH antagonist (cetrorelix or ganirelix, 0.25 mg/day) was initiated to prevent premature ovulation and continued until the trigger day. Ovulation was triggered with a single subcutaneous injection of 250 µg recombinant human chorionic gonadotropin (hCG), or in cases at risk of ovarian hyperstimulation syndrome (OHSS), a GnRH agonist (triptorelin, 0.2 mg) was used instead. Oocyte retrieval was performed 34–36 h post-trigger under ultrasound guidance. GnRH agonist was used only for OHSS-risk patients (*n* = 1 in EM group).

Following oocyte retrieval, cumulus-oocyte complexes were carefully separated from the follicular fluid using a sterile pipette in a medium-free environment to avoid dilution with culture media. The remaining follicular fluid was then centrifuged at 14,000 g for 20 min at 4 °C to remove granulosa cells, blood cells, and any potential debris. To minimize blood contamination, 124 follicles were aspirated with a single-lumen needle under ultrasound guidance, avoiding visible blood vessels, and samples with visible hemolysis were discarded. The clarified supernatant was transferred to sterile tubes and immediately stored at −80 °C to preserve sample integrity for subsequent high-performance liquid chromatography-mass spectrometry (HPLC-MS) analysis. All oocytes in our study were fertilized using conventional IVF procedures.

### Metabolite extraction and detection

The follicular fluid samples were thawed at 4 °C, and 100 μL aliquots were mixed with 400 μL of cold methanol/acetonitrile (1:1, v/v) to precipitate proteins. The mixture was centrifuged for 15 min at 14,000 g and 4 °C. The clear supernatant was then dried using a vacuum centrifuge. For liquid chromatography-mass spectrometry (LC-MS) analysis, the dried metabolites were reconstituted in 100 μL of acetonitrile/water (1:1, v/v) solvent. The metabolomic analysis was conducted using an ultra-high-performance liquid chromatography (UHPLC) system (1290 Infinity LC, Agilent Technologies, Santa Clara, CA, USA) coupled with a quadrupole time-of-flight mass spectrometer (TripleTOF 6600, AB Sciex, Framingham, MA, USA). For separation, a 2.1 mm ×100 mm UPLC column was employed, and gradient elution was performed with appropriate mobile phases for both HILIC and RPLC modes. The electrospray ionization (ESI) source conditions included an ion source temperature of 600 °C and a voltage of ± 5,500 V. Data acquisition was performed in both MS and tandem mass spectrometry (MS/MS) modes over an m/z range of 60–1,000 Da,with parameters optimized for sensitivity and accuracy.

### Quality control and analytical conditions

Quality control (QC) samples were prepared following standard untargeted metabolomics protocols by pooling equal aliquots from all study samples across the four infertility groups. The purpose of using pooled QCs is not to serve as biological controls, but rather to generate a representative, homogeneous reference sample that reflects the overall metabolite composition of the entire cohort.

These pooled QC samples were interspersed regularly throughout the analytical sequence to monitor the instrumental stability, analytical reproducibility, and potential signal drift during data acquisition. This approach is widely adopted and well-documented in untargeted LC–MS metabolomics studies to ensure the reliability and comparability of metabolomic data across the full run (Broeckling et al., 2023; Mosley et al., 2024). Chromatographic separation was performed using an Agilent 1290 Infinity LC system coupled with an AB Sciex TripleTOF 6600 mass spectrometer. For positive ion mode analysis, ESI conditions included a source temperature of 600 ^∘^C and a spray voltage of ± 5,500 V. Data were acquired in both MS and MS/MS modes, with a scan range of 60–1,000 m/z and an accumulation time of 0.2 s per spectrum.

### Data processing and statistical analysis

The raw MS data were converted to MzXML files using ProteoWizard MSConvert (v3.0) and processed with XCMS (v3.16.0) for peak detection and grouping using optimized parameters. Isotopes and adducts were annotated using CAMERA (v1.50.0), and variables with more than 50% nonzero measurements in at least one group were retained. Metabolites were identified by matching m/z values (<10 ppm) and MS/MS spectra with an in-house database. Missing data were imputed using the K-nearest neighbors method, extreme values were excluded, and the total peak area was normalized to ensure comparability across samples.

In this study, all statistical analyses were conducted using R (v4.4). Baseline characteristics were analyzed with Table S1 (v1.4.3), summarizing categorical data as frequencies and percentages and continuous data as medians and interquartile ranges (IQR). Group comparisons were performed using chi-square tests for categorical data and Kruskal–Wallis H tests for continuous data. Metabolomic data and metadata were integrated into a Seurat object. Principal component analysis (PCA) using Seurat (v5.0) and orthogonal partial least squares discriminant analysis (OPLS-DA) using ropls (v1.38) were applied to assess metabolic differences. Differentially abundant metabolites were identified with DESeq2 (v1.46) and visualized through volcano plots (scRNAtoolVis v1.0) and heatmaps (ComplexHeatmap v2.22). Pathway enrichment analyses were performed with Metabo AnalystR (v4.0).

Correlations between metabolites and clinical outcomes were analyzed using corrr (v0.44). Clinical outcomes included follicle development rate (FDR, defined as the number of follicles greater than 14 mm on the trigger day divided by the total number of antral follicles on stimulation day 0), oocyte maturation rate (OMR, defined as the number of metaphase II oocytes divided by the total number of retrieved oocytes), normal fertilization rate (NFR, defined as the number of normally fertilized oocytes with two pronuclei divided by the number of mature oocytes), and high-quality embryo rate (HQER). The HQER was defined as the number of embryos meeting specific morphological criteria on day 3 divided by the total number of fertilized oocytes. High-quality embryos were classified based on the Gardner and Schoolcraft grading system, requiring 6–8 evenly sized, symmetrical blastomeres with ≤10% fragmentation and no multinucleation, assessed under an inverted microscope by two independent embryologists. Interrelations among metabolites were visualized using igraph (v2.1.2). Finally, predictive modeling and feature importance analysis were conducted using randomForest (v4.7).

## Results

### Metabolomic profiling of follicular fluid across different etiologies of female infertility

**Figure 1:**
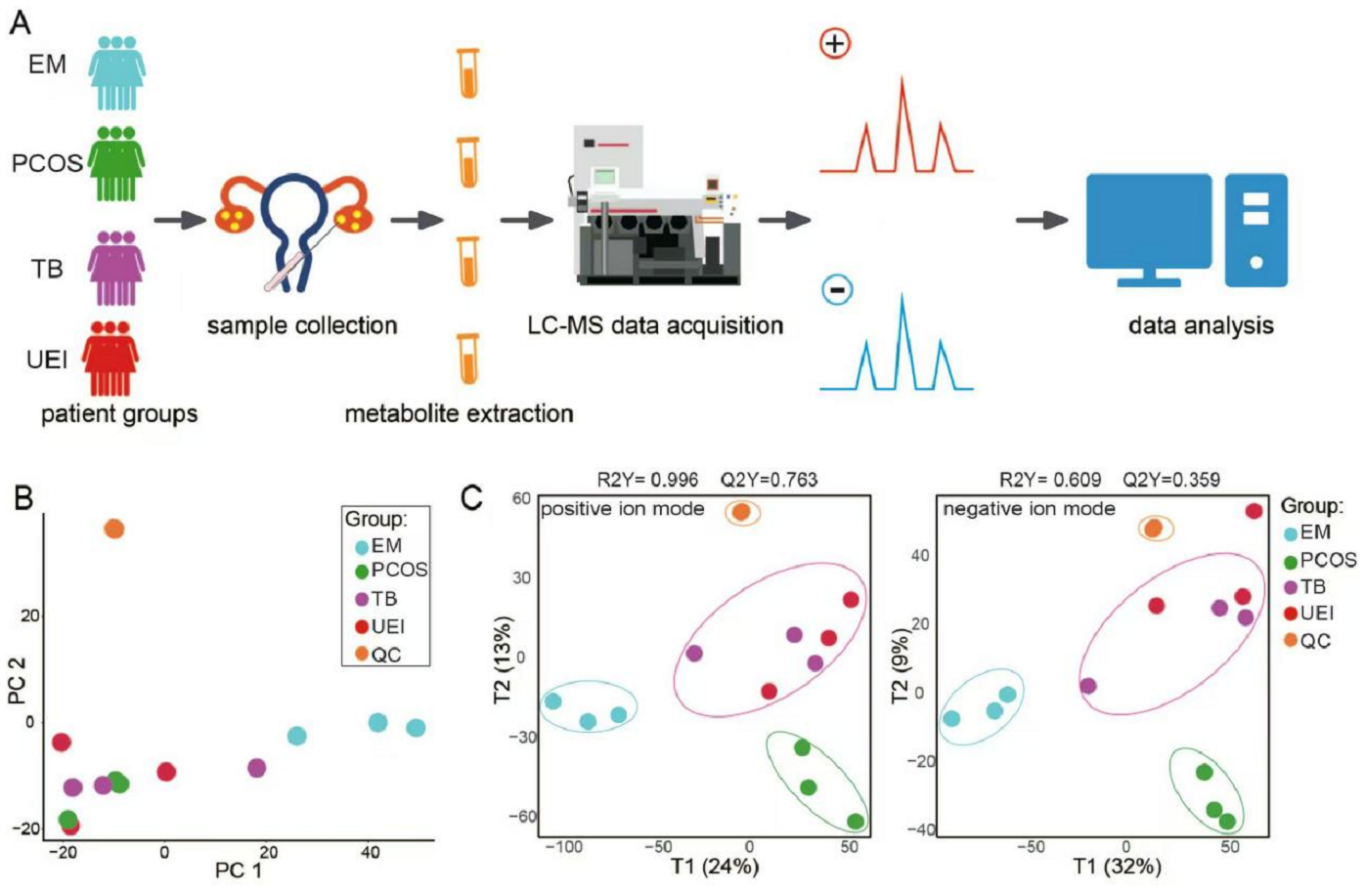
Metabolomic analysis of follicular fluid in female infertility of different etiologies. (A) Experimental design and data acquisition process for metabolomic analysis. (B) Principal component analysis (PCA) of metabolomic profiles in positive Modes. (C) Orthogonal partial least squares discriminant analysis (OPLS-DA) of metabolomic data in positive (left) and negative (right) ion modes. EM, Endometriosis; PCOS, Polycystic Ovary Syndrome; TB, Tubal Blockage; UEI, Unexplained Infertility; QC, Quality Control.

This study investigated the metabolic foundations of female infertility across four etiologies (EM, PCOS, TB, and UEI) by collecting follicular fluid samples, extracting metabolites, and performing LC-MS-based metabolomic profiling. Subsequent data analysis (including PCA and OPLS-DA) revealed distinct metabolic signatures as summarized in Fig. 1A. Principal component analysis (PCA) was applied to the metabolomic data to reduce dimensionality and visualize the distinction among the various infertility groups. The PCA plot clearly differentiated the quality control (QC) samples from the patient samples, with the first two principal components (PC1 and PC2) capturing significant variance, highlighting marked metabolomic distinctions. Notably, EM samples were well-separated from other etiological groups, whereas PCOS, TB, and UEI did not show clear segregation using PCA (Fig. 1B). Consequently, we employed orthogonal partial least squares discriminant analysis (OPLS-DA) for a more targeted dimension reduction and supervised analysis to better differentiate between these overlapping groups. The OPLS-DA results demonstrated robust model fit statistics in the positive model, evidenced by high R2Y and Q2Y values, affirming the reliability of the observed metabolomic distinctions. In both positive and negative ion modes, the OPLS-DA score plots revealed well-defined clusters corresponding to each patient group. Notably, the metabolic profiles of the EM and TB groups were particularly distinct, setting them apart markedly from other groups (Fig. 1C). Interestingly, both PCA and OPLS-DA analyses revealed that a sample from the TB group closely aligned with the EM cluster (Figs. 1B–1C). In summary, metabolomic profiling of follicular fluid reveals distinct signatures that can distinguish between different causes of female infertility.

### Characterization of metabolite profiles and pathway enrichment across different infertility etiologies

**Figure 2:**
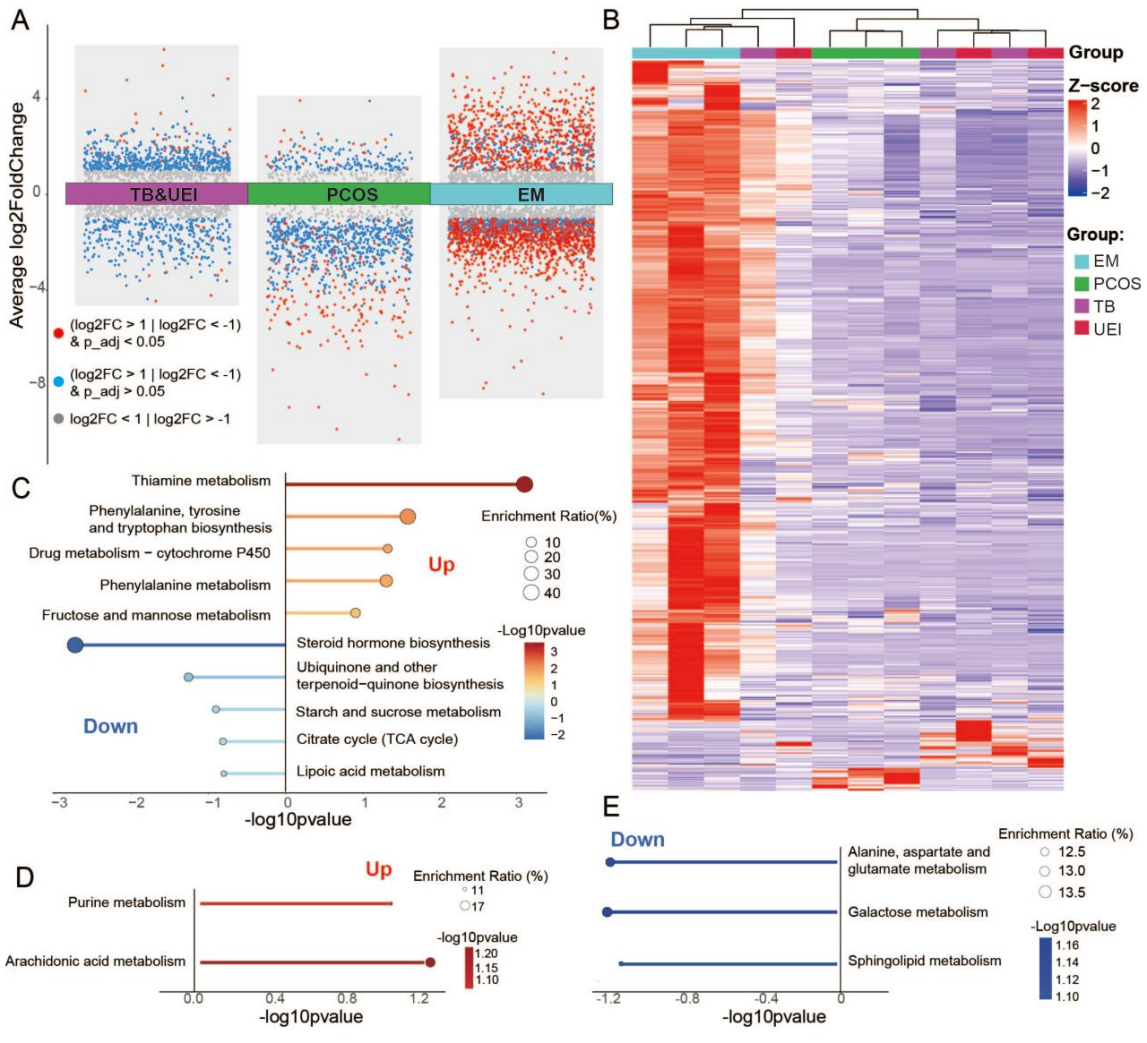
Metabolomic analysis and pathway enrichment in follicular fluid across different infertility etiologies. (A) Volcano plot of metabolite concentrations in positive ion mode across patient groups. (B) Heatmap visualization of significantly altered metabolites (log2FC > 1, *p*_*a*_*dj* < 0.05) across patient groups. (C) Pathway enrichment analysis for the endometriosis group (D) Pathway enrichment analysis for tubal blockage and unexplained infertility groups (E) Pathway enrichment analysis for polycystic ovary syndrome (PCOS) group. EM, Endometriosis; PCOS, Polycystic Ovary Syndrome; TB, Tubal Blockage; UEI, Unexplained Infertility.

Our subsequent analyses employed positive ion mode metabolomic data, which was demonstrated to effectively differentiate patient groups in earlier OPLS-DA results. Using the Seurat analysis tool, we compared the metabolite concentration differences across various infertility patient groups and visualized these differences using volcano plots. The plots revealed notable heterogeneity in metabolite changes among the infertility groups. In the EM group, many metabolites showed significant increases and decreases, as evidenced by numerous red points in both the upregulated and downregulated areas. In contrast, the TB and UEI group showed some significantly elevated metabolites, while the PCOS group exhibited a higher number of decreased metabolites relative to the TB and UEI group (Fig. 2A).

Following the volcano plot analysis, we presented a heatmap that illustrates the metabolites significantly altered among the different infertility groups, identified by Times New Roman−0.05pt12ptlog2 fold changes exceeding 1 and adjusted *p*-values below 0.05. This heatmap confirms that individuals within the same patient group share similar and distinct metabolic signatures compared to other groups. Interestingly, we observed that a patient from the TB and UEI groups exhibited metabolic features similar to those in the EM group, which aligns with our PCA findings where a patient from the TB group closely clustered with the EM group (Fig. 2B).

After identifying differentially concentrated metabolites, we conducted pathway enrichment analysis to elucidate the specific disruptions for each infertility group. In the EM group, pathways such as the tricarboxylic acid (TCA) cycle and steroid hormone biosynthesis were notably enriched due to decreased metabolite concentrations, indicating disruptions in energy production and hormonal regulation. Conversely, pathways like thiamine metabolism were enriched with increased metabolites, highlighting enhanced activity in these areas (Fig. 2C). In the PCOS group, key pathways like purine and arachidonic acid metabolism showed a decrease in metabolites, impacting ovulation processes (Fig. 2D). For the TB and UEI groups, pathways involved in inflammation and metabolism were enriched with increased metabolites, highlighting the metabolic basis of these conditions (Fig. 2E).

In summary, different patient groups exhibit distinct metabolic profiles, with significant metabolite differences enriched in various pathways, offering potential insights into their underlying mechanisms.

### Metabolomic correlations with follicular development and embryo quality

**Figure 3:**
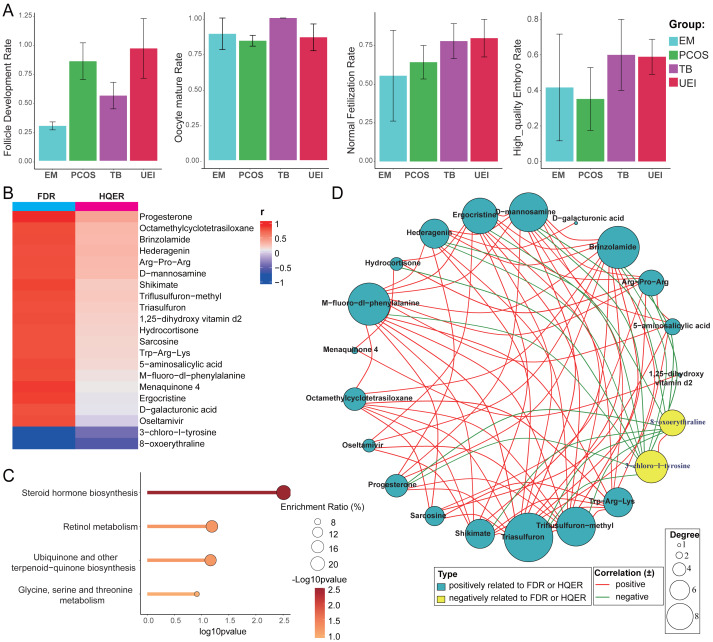
Metabolomic insights into follicular development and embryo quality across infertility etiologies. (A) Comparison of follicle development rate (FDR), oocyte maturation rate (OMR), fertilization rate (FR), and high-quality embryo formation rate (HQER) across patient groups. (B) Metabolites significantly correlated with FDR and HQER, identified by correlation coefficients > 0.8 or < −0.8 and *p*-values < 0.05. (C) Pathway enrichment analysis of selected metabolites, highlighting key pathways such as steroid hormone biosynthesis and retinol metabolism. (D) Correlation network of the selected metabolites, demonstrating interrelationships among metabolites influencing FDR and HQER. EM, Endometriosis; PCOS, Polycystic Ovary Syndrome; TB, Tubal Blockage; UEI, Unexplained Infertility.

To investigate the impact of different infertility etiologies on follicular development and associated outcomes, we compared follicle development rate (FDR), oocyte maturation rate, fertilization rate, and high-quality embryo formation rate (HQER) across the EM, PCOS, TB, and UEI groups. The results revealed a significantly lower FDR in the EM group compared to other groups, while HQER in the EM and PCOS groups was slightly lower than in the TB and UEI groups. Notably, there were no significant differences in oocyte maturation and fertilization rates among the groups. Interestingly, a reduction in FDR was also observed in the TB group. Upon further inspection of the raw data, this reduction was attributed to the previously identified TB patient whose metabolomic profile closely resembled that of the EM group and exhibited a notably lower FDR (Fig. 3A).

Given the distinct metabolic profiles observed in the EM group and their significantly lower follicle development rate (FDR) compared to other groups, we performed a correlation analysis between all detected metabolites and FDR. Metabolites with correlation coefficients >0.8 or <−0.8 and *p*-values <0.05 were further analyzed for their correlation with high-quality embryo rate (HQER). Among the identified metabolites, 19 showed strong positive correlations with FDR but had relatively weak correlations with HQER. Two metabolites, 3-chloro-l-tyrosine and 8-oxoerythraline, were negatively correlated with both FDR and HQER, highlighting their potential detrimental roles (Fig. 3B).

We also analyzed the interrelations among the selected metabolites. Pathway enrichment analysis of the positively correlated metabolites revealed strong associations with key pathways such as steroid hormone biosynthesis and retinol metabolism (Fig. 3C), which have been previously reported in the literature as key contributors to follicular growth and maturation. Metabolites that showed significant positive correlations with FDR were also predominantly positively correlated with each other, suggesting a cooperative network supporting follicular development. Conversely, the two metabolites negatively correlated with FDR, 3-chloro-l-tyrosine and 8-oxoerythraline, exhibited significant negative correlations with the majority of other metabolites,highlighting their potential antagonistic roles (Fig. 3D). These findings above highlight the distinct metabolic profiles associated with infertility etiologies, particularly in the EM group, where disrupted pathways like steroid hormone biosynthesis and retinol metabolism are closely linked to reduced follicular development.

### Metabolite-based prediction of endometriosis

**Figure 4:**
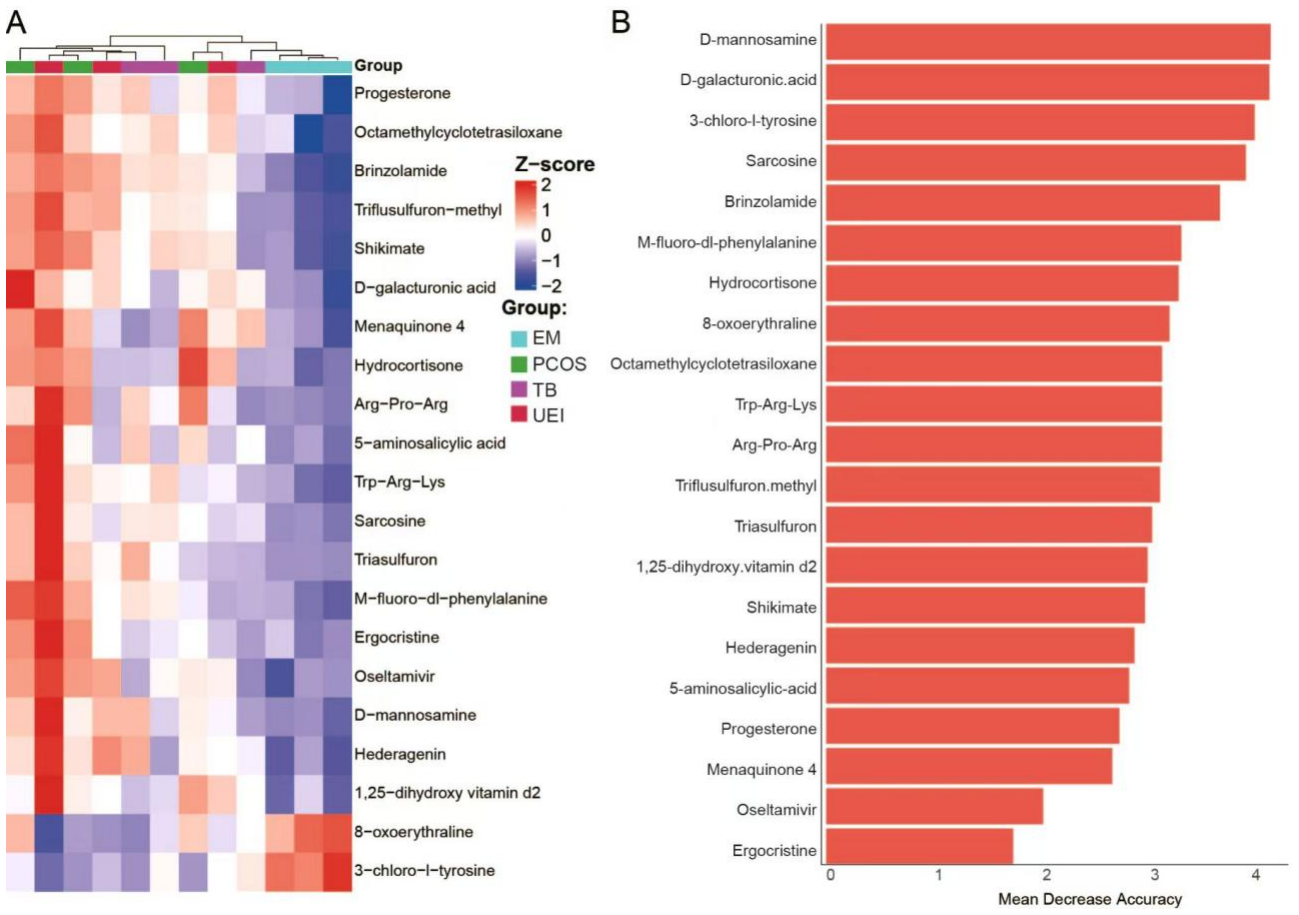
Metabolite concentrations and predictive modeling for endometriosis. (A) Heatmap showing the concentrations of metabolites significantly correlated with follicle development rate (FDR) across all patient groups. (B) Feature importance plot for the predictive model of endometriosis (EM).

To evaluate the diagnostic potential of metabolites identified in Fig. 3B, their concentrations were examined across all samples. Notably, metabolites positively correlated with follicle development rate (FDR), such as D-mannosamine and D-galacturonic acid, showed lower concentrations in the endometriosis (EM) group and in the TB sample with metabolic similarities to EM, as compared to other groups. Conversely, metabolites negatively correlated with FDR, including 3-chloro-l-tyrosine and 8-oxoerythraline, exhibited higher concentrations in these samples, further supporting their potential roles as biomarkers for EM (Fig. 4A). Using these metabolites, we constructed a predictive model to classify EM samples. The model identified key metabolites contributing to the classification, with variables such as D-mannosamine, D-galacturonic acid, and 3-chloro-l-tyrosine displaying the highest importance (Fig. 4B). The prediction model demonstrates strong potential for distinguishing EM from other infertility etiologies based on metabolic profiles, providing a foundation for future diagnostic development.

## Discussion

This study conducted metabolomic profiling of follicular fluid to distinguish the metabolic characteristics of patients with endometriosis (EM), polycystic ovary syndrome (PCOS), and tubal blockage (TB). EM patients exhibited distinct metabolomic profiles compared to PCOS and TB groups, which showed more similar characteristics. Notably, metabolites related to thiamine metabolism were elevated, while those involved in steroid hormone metabolism were significantly reduced in EM patients. Additionally, follicle development rate (FDR) and high-quality embryo rate (HQER) were markedly lower in the EM group. Metabolites highly correlated with FDR were enriched in the steroid hormone pathway, with significantly lower concentrations in EM patients. These findings highlight the pivotal role of follicular fluid metabolites in identifying EM and predicting IVF outcomes, offering new insights for optimizing reproductive strategies in EM patients.

The causes of female infertility are diverse, including both tubal and ovarian factors (Penzias et al., 2021). Ovarian factors are particularly complex, involving follicular development disorders and ovulation abnormalities, requiring tailored treatment strategies. In this context, metabolomics, as a high-throughput technology, offers a precise reflection of the follicular microenvironment, aiding in understanding these mechanisms (Mehrparavar et al., 2019). Using the OPLS-DA model, we distinguished EM patients from those with PCOS and TB, identifying distinct metabolic profiles in follicular fluid. Interestingly, one patient from the TB group exhibited metabolic features similar to the EM group and was clustered with them.

Further analysis suggested this patient might have early-stage EM, as ovarian cysts were detected on ultrasound despite the absence of a pathological diagnosis. These findings highlight the potential of follicular fluid metabolites as a tool for early EM diagnosis in patients undergoing IVF, offering a less burdensome alternative to surgical confirmation. Future studies should explore their correlation with blood metabolites to develop broader, less invasive diagnostic approaches for early detection and management of EM.

Our pathway enrichment analysis showed that upregulated metabolites in the follicular fluid of EM patients were mainly enriched in thiamine, phenylalanine, tyrosine, and fructose pathways. Active phenylalanine metabolism may indicate increased demand for aromatic amino acids related to stress response, signal transduction, or hormone metabolism (Ennis et al., 2020; Qi et al., 2019), while elevated phenylalanine and fructose metabolism can generate reactive oxygen species, raising oxidative stress levels (Chen et al., 2023; Wu et al., 2022). This necessitates support from antioxidants like thiamine to maintain redox balance. Previous studies have shown that ovarian EM induces inflammation and oxidative stress in follicular fluid, with elevated levels of inflammatory cytokines such IL-8, and IL-12 and upregulated NF*κ*B signaling in granulosa cells, exacerbating these effects (Choi et al., 2015; Gschwandtner, Derler & Midwood, 2019; Li et al., 2023; Liu et al., 2013). In addition, EM patients show higher oxidative stress markers (*e.g.*, reactive oxygen species) and lower antioxidant levels (*e.g.*, glutathione), potentially contributing to granulosa cell aging and ovarian dysfunction (Luo et al., 2024).

In contrast, follicular fluid from patients with tubal-factor infertility showed minor enrichment in purine metabolism pathways. In PCOS patients, downregulated metabolites were primarily enriched in galactose and sphingolipid metabolism pathways. Reduced galactose metabolism indicates impaired energy utilization, while alterations in sphingolipid metabolism may lead to membrane instability and hinder signal transduction, negatively impacting cell proliferation,differentiation, and apoptosis regulation (Delwing-de Lima et al., 2023; Ogretmen, 2018). PCOS is strongly associated with metabolic abnormalities and insulin resistance, which may explain the metabolic imbalances in galactose and sphingolipid metabolism, thereby affecting follicular maturation (Li et al., 2019). These findings provide important insights into follicular development and the pathogenesis of PCOS.

Unlike previous studies comparing single etiologies to controls, our direct multi-group analysis revealed that metabolites related to the steroid biosynthesis pathway were significantly reduced in EM patients, shedding light on the mechanism underlying infertility in EM. Steroid hormone synthesis, crucial for oocyte maturation and follicular growth, relies on cholesterol and enzymatic activity to produce hormones like estrogen and progesterone (Miller & Auchus, 2011; Zheng et al., 2023). The significant reduction of steroid biosynthesis-related metabolites in EM patients highlights a disruption in hormone synthesis, leading to lower follicle development rates (FDR) despite similar fertilization and embryo quality rates across groups. Interestingly, a TB patient with metabolic features resembling EM also exhibited a low FDR, further supporting the critical role of this pathway in follicular development. Correlation analysis identified 18 metabolites strongly associated with FDR, primarily enriched in the steroid biosynthesis pathway, confirming their critical role. Furthermore, two metabolites, 3-chloro-L-tyrosine and 8-oxoerythraline, were negatively correlated with FDR and significantly elevated in EM patients. These metabolites, associated with oxidative stress and inflammation, may antagonize steroid biosynthesis metabolites. Notably, 3-chloro-L-tyrosine is an oxidative stress and inflammation marker, while 8-oxoerythraline is linked to neuronal inflammation and DNA Repair (Fleszar et al., 2020; Seßenhausen et al., 2023; Setti-Perdigão et al., 2013; Umihara et al., 2016).

To advance these findings, we also developed a predictive model identifying D-mannosamine, D-galacturonic acid, and 3-chloro-L-tyrosine as potential markers for EM. These results, while limited by sample size, suggest integrating follicular fluid and blood metabolite data could enable diagnosis that does not require additional invasive procedures, offering a more precise diagnostic tool for patients.

This study has several limitations. The relatively small sample size inevitably limits the statistical power and generalizability of our findings; however, we carefully applied rigorous quality control and statistical analyses to ensure data reliability. Additionally, we did not include a healthy control group due to ethical and practical constraints, prioritizing instead a comparative analysis across four infertility etiologies within a unified framework. Variations in ovulation triggers represent a limitation, though clinically necessary for patient safety. Despite these limitations, our study provides novel insights into etiology-specific metabolic alterations and serves as a pilot investigation to guide future large-scale and multicenter studies.

In summary, this study systematically compared follicular fluid metabolic profiles across different infertility etiologies and identified distinctive alterations in steroid biosynthesis and thiamine metabolism pathways in EM patients. The discovery of EM-specific biomarkers, such as 3-chloro-L-tyrosine, provides new insights into the underlying mechanisms of EM-related infertility and M-offers a potential framework for developing non-invasive diagnostic tools and personalized treatment strategies to improve reproductive outcomes.

## Supplemental Information

10.7717/peerj.20786/supp-1 Supplemental Information 1 Baseline Characteristics of Participants by Group

10.7717/peerj.20786/supp-2 Supplemental Information 2 Metabolite status

10.7717/peerj.20786/supp-3 Supplemental Information 3 NEG data

10.7717/peerj.20786/supp-4 Supplemental Information 4 POS data

## Additional Information and Declarations

### Data Availability

The following information was supplied regarding data availability: The dataset is available at Zenodo: Wei, F. (2025). Metabolomic Profiling of Follicular Fluid Reveals Unique Pathways in Endometriosis and Infertility Etiologies-a pilot study. https://doi.org/10.5281/zenodo.17058435.
