# Supplementary material for: Metabolomic profiling of follicular fluid reveals unique pathways in endometriosis and infertility etiologies: a pilot study
Source: PeerJ. 2026 Feb 10;14:e20786. doi: 10.7717/peerj.20786 (PMC12903890; doi:10.7717/peerj.20786)
Supplement: Supplemental Information 1 [file peerj-14-20786-s001.doc]

### ****Table 1. Baseline Characteristics of Participants by Group****

| Variable | UEI (N=3) | PCOS (N=3) | EM (N=3) | TB (N=3) |
| --- | --- | --- | --- | --- |
| Age (years) | 31.00 ± 1.00 | 35.00 ± 1.00 | 33.33 ± 3.21 | 32.33 ± 2.08 |
| BMI (kg/m²) | 22.33 ± 0.58 | 23.70 ± 4.16 | 28.60 ± 3.56 | 23.13 ± 1.06 |
| AFC | 14.00 ± 3.61 | 13.67 ± 5.51 | 11.33 ± 2.08 | 16.00 ± 7.21 |
| AMH (ng/mL) | 6.03 ± 3.33 | 6.43 ± 3.03 | 4.25 ± 1.26 | 3.41 ± 1.27 |
| FSH (IU/L) | 7.88 ± 2.36 | 5.79 ± 1.96 | 7.48 ± 0.98 | 6.71 ± 0.56 |
| E2 (pg/mL) | 27.63 ± 8.22 | 32.87 ± 16.78 | 19.60 ± 2.65 | 18.93 ± 3.00 |
| P (ng/mL) | 0.63 ± 0.47 | 0.49 ± 0.35 | 0.52 ± 0.23 | 0.50 ± 0.35 |
| T (ng/mL) | 0.32 ± 0.11 | 0.41 ± 0.17 | 0.41 ± 0.16 | 0.41 ± 0.12 |

**EM**: Endometriosis, **PCOS**: Polycystic Ovary Syndrome, **TB**: Tubal Blockage, **UEI**: Unexplained Infertility
